# 10-W pulsed operation of substrate emitting photonic-crystal quantum cascade laser with very small divergence

**DOI:** 10.1186/s11671-015-0877-4

**Published:** 2015-04-14

**Authors:** Dan-Yang Yao, Jin-Chuan Zhang, Olivier Cathabard, Shen-Qiang Zhai, Ying-Hui Liu, Zhi-Wei Jia, Feng-Qi Liu, Zhan-Guo Wang

**Affiliations:** Key Laboratory of Semiconductor Materials Science, Institute of Semiconductors, Chinese Academy of Sciences, P.O. Box 912, Beijing, 100083 China

**Keywords:** 42.55.Px, 42.55.Tv, 42.60.Da, 42.60.Jf, Quantum cascade laser (QCL), Photonic crystal, Substrate emitting, Distributed feedback (DFB), Low divergence angle

## Abstract

High-power broad area substrate emitting photonic-crystal distributed feedback (DFB) quantum cascade lasers (QCLs) emitting around 4.73 μm is reported. Two-dimensional centered rectangular photonic-crystal (CRPC) grating is introduced to enhance optical coherence in large area device. Main lobe far-field radiation pattern with a very small divergence angle of about 0.65° × 0.31° is obtained. A record peak output power for vertical emitting QCLs exceeding 10 W is obtained with high reflectivity (HR) coating. Robust single longitudinal mode emission with a side mode suppression ratio (SMSR) of 30 dB is continuously tunable by the heat sink temperature up to 65°C.

## Background

Recent successive performance breakthroughs of vertical emitting quantum cascade lasers (QCLs) [1-4] have aroused more attention and further research into this field. Due to easy packaging [5], lacking of catastrophic optical damage [6] and high beam quality, vertical emitting QCLs become a prominent candidate as ideal source in mid-infrared spectrum range.

The previous reported vertical emitting QCLs [7-9] were limited to pulsed mode operation with output power below 1 W. Subsequently, with the advent of epilayer-down bonding technology for substrate emitting quantum cascade lasers (a kind of vertical emitting laser with light out from the substrate side, SE-QCLs) [10], continuous wave (CW) operations were demonstrated with second-order distributed feedback (DFB) grating stripe [11,12] and ring [13] cavity laser. However, for some applications, such as remote detection and laser-induced blinding, high pulsed power is more desirable than CW operation. Experiments [14] have verified the positive correlation between power and emitting width for edge emitting QCLs. Similarly, 60- and 100-μm-wide second-order DFB SE-QCLs emitting peak power of 1.8 and 2.4 W at room temperature were achieved respectively [15], while simply increasing the width of devices leads to degraded spatial beam properties [14]. Two-dimensional (2D) photonic-crystal (PC) DFB grating is a periodic structure with great potential, which is not only compatible to a large gain medium size but also providing effective modulation of longitudinal mode (spectrum) and transverse mode (far-field in transverse direction) simultaneously [16-18]. Photonic crystal vertical emitting QCLs were designed to produce vertical radiation from rectangle aperture (200 μm × 200 μm) with narrow divergence angle of 2.4° × 1.8° [2].

In this paper, we present a promising approach based on 2D centered rectangular photonic-crystal (CRPC) theory to fabricate broad area substrate emitting mid-infrared quantum cascade laser. A compromised cross angle *θ* for CRPC lattice is chosen to enhance the coupling strength in the transverse direction without degradation of single longitudinal mode performance after numerical simulation. At room temperature, the laser emits total optical power as high as 10 W with a very small divergence far-field angle below 1° in two directions.

## Methods

### Structure and simulation

**Figure 1:**
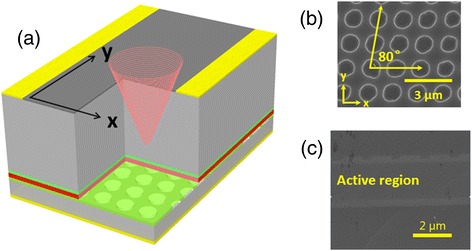
3D sketch, SEM, and cross section of the lattice. **(a)** Three-dimension (3D) sketch of a device. **(b)** Scanning electron microscope (SEM) image for centered rectangular photonic-crystal grating. **(c)** Cross section of the lattice after MOCVD regrowth.

Figure 1a shows three-dimension (3D) sketch of the substrate emitting device. The active region of QCL structure is based on strain compensated In_0*.*67_Ga_0*.*33_As/In_0*.*36_Al_0*.*64_As quantum wells and barriers, identical with ref. [19]. A 1.54-μm active region is sandwiched between two 300-nm-thick InGaAs layers grown by solid source molecular beam epitaxy (MBE). The CRPC structure with cross angle *θ* of 80° (Figure 1b) is defined on the upper InGaAs layer using double-exposure holographic lithography (DEHL) technique [20]. Then, 3.0 μm InP cladding layer and 0.8 μm high-doped InP contact layer are grown by metal organic chemical vapor deposition (MOCVD). In our design, embedded CRPC lattice structure between the high-index InGaAs layer and low-index InP cladding, as shown in Figure 1c, is adopted in order to get the waveguide loss smaller than surface metallic lattice structure because the latter will introduce heavily metallic ohmic losses at the interface between metal and semiconductor.

2D photonic-crystal geometry obtains vertical coherent oscillation radiation over a broad area based on the band edge effect [21] with an increased optical path length, which leads to a gain enhancement. Typical square and hexagonal lattice have been principally investigated for years. As a novel structure, centered rectangular crystalline geometry attracted increasing attention [22,23] since it may have potential for a combination of advantages from square and hexagonal lattice. In order to make a comparison among the three types of PC structure and obtain optimized design parameters in our vertical emitting QCLs, we analyze the band structure and coupling coefficient of different types of 2D photonic-crystal.

**Figure 2:**
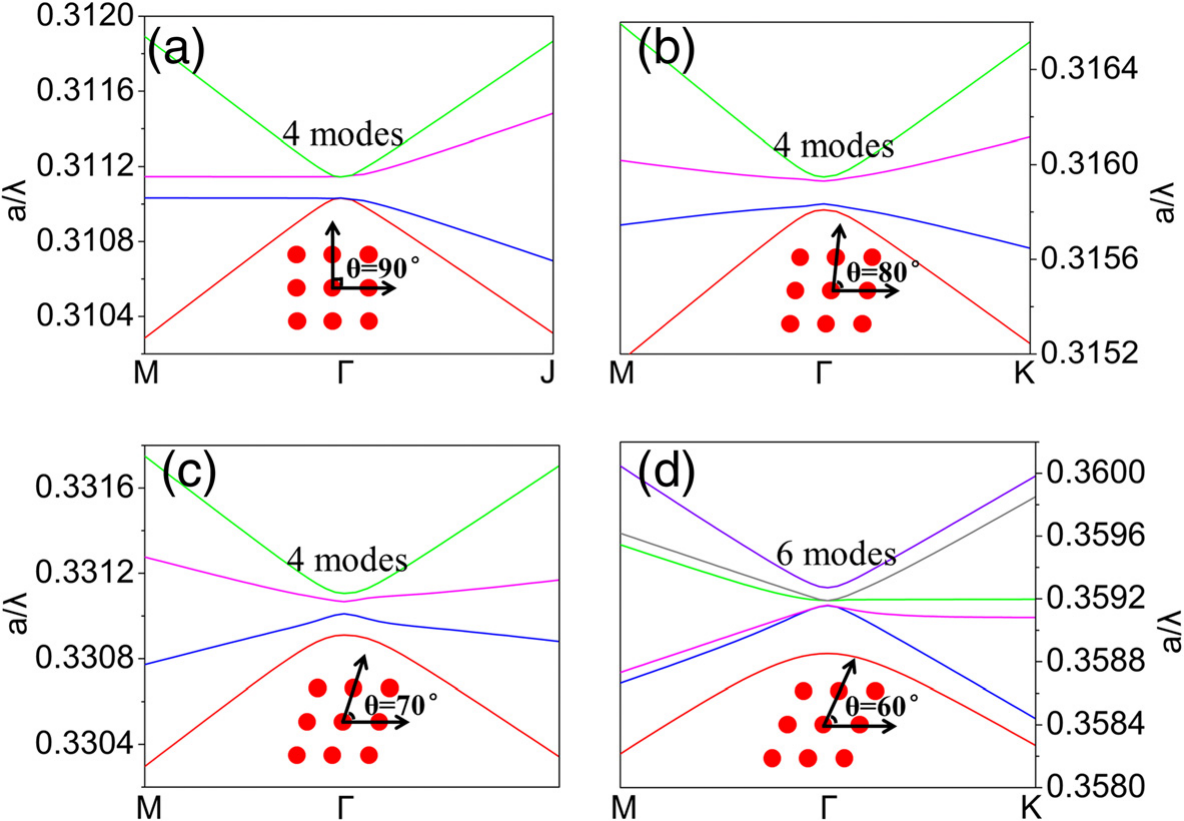
Band structure near the *Γ* point of lattice PC. With cross angle *θ* ranging from **(a)** 90°, square lattice. **(b)** 80° and **(c)** 70°, in turn, centered rectangular lattice. **(d)** 60°, hexagonal lattice.

By using bandsolve module of Rsoft, based on 2D plane wave (PW) expansion method [24], we first analyze the band structure for different types of lattice and calculate the number of band edge longitudinal modes simultaneously. Figure 2 shows the enlarged band structure near the *Γ* point of lattice PC with cross angle *θ* ranging from 90° to 60° in a step of 10°, and the insets at the bottom of each picture depict the pattern of different types of photonic-crystal. The number of band edge resonant fundamental longitudinal mode depends on the lattice structure. There are both four modes for square (Figure 2a) and centered rectangular lattice (Figure 2b,c) while six modes presented for hexagonal lattice (Figure 2d). It indicates that square and centered rectangular lattices are more likely to realize stable single longitudinal mode oscillation.

**Figure 3:**
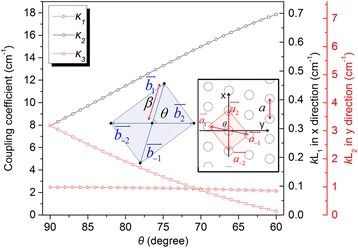
Coupling coefficients *κ*_1_, *κ*_2_, and *κ*_3_ vs. lattice cross angle *θ* from 90° to 60°. The refractive index contrast Δ*n* = 0.008. Inset: Right, centered rectangular lattice PC in real space with lattice constant *a* and lattice cross angle *θ*. Left, reciprocal lattice space corresponding to the real space with lattice constant *β*.

We next investigate the impact of cross angle *θ* on the in-plane coupling coefficient. Given the chosen exposure period *a*_0_ and fixed radius of cylinder *r*, the coupling coefficients only change with the lattice cross angle *θ*. In the inset of Figure 3, the primitive translation vectors labeled ${\overset{\rightharpoonup }{a}}_1=a\left(0,1\right),\kern0.24em {\overset{\rightharpoonup }{a}}_2$, = *a*(−sin *θ*, cos *θ*) and the primitive reciprocal lattice vectors labeled ${\overset{\rightharpoonup }{b}}_1=l\beta \left( \cos \theta, \sin \theta \right),\kern0.24em {\overset{\rightharpoonup }{b}}_2$, = *mβ*(−1, 0) where, $a=\frac{a_0}{ \sin \theta }$, $\beta =\frac{2\pi }{a_0}$

We calculate the coupling coefficients by using the following expression [25]:

1 $$ {\kappa}_1=\frac{2\pi \varDelta n}{\lambda}\frac{1}{A}{\displaystyle \int }{\displaystyle \int } dxdz \exp \left[-i\left(\overset{\rightharpoonup }{b_1}-\overset{\rightharpoonup }{b_{-1}}\right)\cdot r\right] $$

2 $$ {\kappa}_2=\frac{2\pi \varDelta n}{\lambda}\frac{1}{A}{\displaystyle \int }{\displaystyle \int } dxdz \exp \left[-i\left(\overset{\rightharpoonup }{b_1}-\overset{\rightharpoonup }{b_2}\right)\cdot r\right] $$

3 $$ {\kappa}_3=\frac{2\pi \varDelta n}{\lambda}\frac{1}{A}{\displaystyle \int }{\displaystyle \int } dxdz \exp \left[-i\left(\overset{\rightharpoonup }{b_1}-\overset{\rightharpoonup }{b_{-2}}\right)\cdot r\right] $$

For (*l* = *m* = 2) coupling order:

$$ \overset{\rightharpoonup }{b_1}-\overset{\rightharpoonup }{b_{-1}}=\overset{\rightharpoonup }{b_2}-\overset{\rightharpoonup }{b_{-2}}=2\sqrt{l^2+{m}^2}\beta =4\beta, \kern0.24em \overset{\rightharpoonup }{b_1}-\overset{\rightharpoonup }{b_2}=l\beta \sin \frac{\theta }{2}=2\beta \sin \frac{\theta }{2},\kern0.24em \overset{\rightharpoonup }{b_1}-\overset{\rightharpoonup }{b_{-2}}=m\beta \cos \frac{\theta }{2}=2\beta \cos \frac{\theta }{2}, $$

where *A* is the area of the primitive cell of the reciprocal lattice, refractive index contrast Δ*n* = 0.008 derived from 1D effective index method [26], *a*_0_ = 1.49 μm, and *r* = 0.5 μm.

The coefficient *κ*_1_ accounts for distributed reflection-like diffraction into the counter propagating in-plane wave, such as from $\overset{\rightharpoonup }{b_1}$ into $\overset{\rightharpoonup }{b_{-1}}$ or from $\overset{\rightharpoonup }{b_2}$ into $\overset{\rightharpoonup }{b_{-2}}$. The coefficient *κ*_2_ and *κ*_3_ represent diffraction into the oblique in-plane wave vectors $\overset{\rightharpoonup }{b_1}$ into $\overset{\rightharpoonup }{b_2}$ and $\overset{\rightharpoonup }{b_1}$ into $\overset{\rightharpoonup }{b_{-1}}$. Figure 3 shows the calculated coupling coefficient *κ*_1_, *κ*_2_, and *κ*_3_ as a function of lattice cross angle *θ*. We find that when *θ* changes from 90° to 60°, *κ*_1_ slightly reduces from 2.47 to 2.14 cm^−1^. Being different from *κ*_1_, the values of *κ*_2_ and *κ*_3_ are identical 7.96 cm^−1^ at the beginning; then, *κ*_2_ rapidly increases to 17.86 cm^−1^ and *κ*_3_ reduces to 0.35 cm^−1^. In order to obtain uniform modal intensity distribution throughout the device, the coupling strength *κL*, where *L* is the device length in the coupling direction, must be or close to 1 [27]. This means the centered rectangular lattice, especially for *θ* = 80°, enjoys an enlarged *κ*_2_*L*_1_ (*L*_1_ = 0.039 cm) = 0.45 and reduced *κ*_3_*L*_2_ (*L*_2_ = 0.4 cm) = 1.9 compared to square lattice that 0.3 and 3.2, respectively. Therefore, combining with the preceding analysis of the band structure, vertical emitting laser adopting centered rectangular lattice is a good compromise between square lattice and hexagonal lattice to achieve both single longitudinal mode operation and high efficiency vertical emitting.

### Device fabrication

To make the buried CRPC grating, the top InP cladding was removed down to the upper InGaAs layer. A second-order CRPC lattice was defined on the upper InGaAs layer using DEHL technique and subsequently etched to a depth of 150 nm by wet chemical etching. The first exposure with a grating period of 1.49 μm was set along longitudinal direction, and the second exposure with the same period was oblique with a cross angle *θ* of 80°. The fabrication of our devices presented in this paper started from a CRPC lattice MOCVD regrowth ready QCL wafer. The sample was firstly processed into ridge mesa waveguide with a width of 390 μm. Then, the following processing procedures were similar to the description in ref. [11]. Finally, the wafer was cleaved into 4-mm-long bars with edge facet high reflective (HR) and substrate facet anti-reflective (AR) coating. For testing, the laser bars were mounted epilayer down on SiC submounts with indium solder, which were subsequently soldered on copper heat sinks.

## Results and discussions

Device testing was done on an automatic temperature control stage. For electro-optical characterization, the substrate emitting output power was measured with a calibrated thermopile detector that collected laser radiation. The lasing spectra measurement was performed using a Fourier transform infrared spectrometer with a resolution of 0.125 cm^−1^ in rapid scan mode. 2D far-field was done by placing a mercury cadmium telluride (MCT) detector on a 2D stepped motor control translation stage (minimum step of 100 μm, maximum scans range of 4.5 × 4.5 cm), placed at 25 cm away from the laser. After collecting lock-in amplification of the detector signal from the far-field, we processed the data with distance coordinate into angle coordinate.

**Figure 4:**
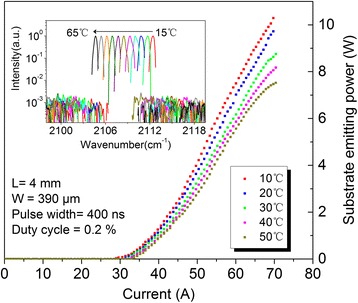
Power versus current (*L*-*I*) characterization of a 4**-**mm-long CRPC substrate emitting QCL. With a ridge width of 390 μm at different heat sink temperatures between 10°C and 50°C. The inset shows the semilog plot of single-mode emission spectra at an injection current of 1.1*I*th under pulsed mode operation, and a SMSR of 30 dB is maintained for the entire temperature range of 15°C to 65°C. Device edge facets were high-reflective coated, and substrate facets were anti-reflective coated.

Our devices realized high-power operation above room temperature in pulsed mode with a pulse width of 400 ns and repetition frequency of 5 kHz. Figure 4 shows the power versus current (*L*-*I*) characterization of a 4-mm-long CRPC substrate emitting QCL with ridge width of 390 μm at different heat sink temperatures. At 10°C, the laser emits up to maximum peak power of 10.3 W with slope efficiencies around 0.38 W/A and threshold current density around 1.86 kA/cm^2^. The maximum wall plug efficiency (WPE) of 1.1% was obtained at a current of 60 A. When the temperature rises up to 50°C, the corresponding value drops to 7.7 W, 0.29 W/A and increases to 2.15 kA/cm^2^. The inset of Figure 4 shows the semilog plot of single-mode emission spectra at an injection current of 1.1*I*th between 15°C and 65°C with a step of 5°C. Single longitudinal mode emission with a side mode suppression ratio (SMSR) of about 30 dB at the entire temperature range is obtained. The peak emission spectrum is observed to shift from 2,112.4 cm^−1^ at 15°C to 2,104.6 cm^−1^ at 65°C, corresponding to a temperature tuning coefficient ∇*v*/∇*T* = −0.155 cm^−1^ C^−1^.

**Figure 5:**
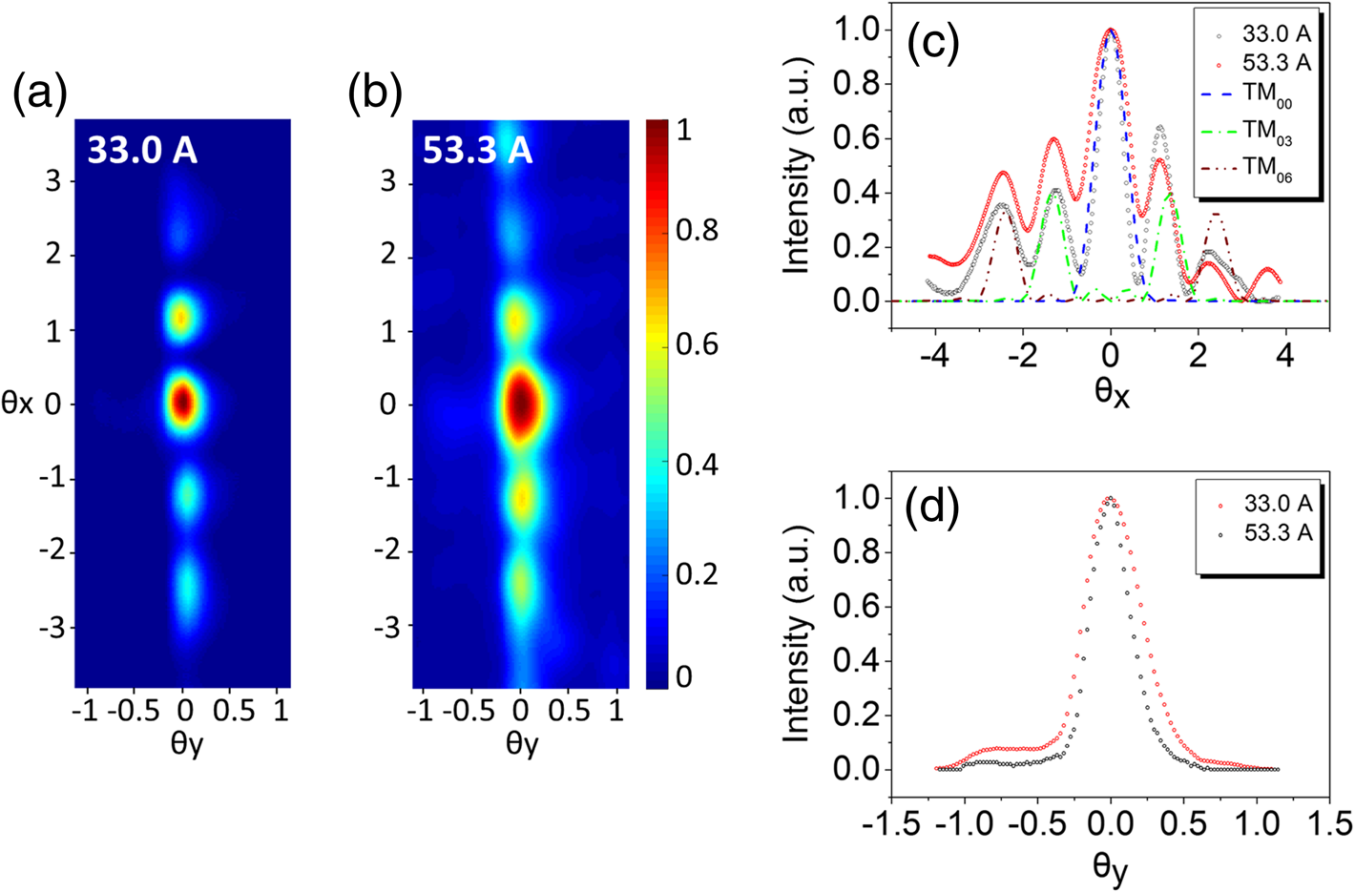
Measured far-field intensity distributions for the substrate emitting device. **(a)** 2D far-field intensity distribution under pulsed driving current of 33.0 A. **(b)** 53.3 A. **(c)** Measured 1D far-fields for *θx* correspond to **(a)** and **(b)** for comparison. Simulated results of TM_00_, TM_03_, and TM_06_ are depicted using dotted lines **(d)** 1D far-fields for *θy*.

Providing that the fundamental transverse mode (TM_00_) has priority to lase, vertical emitting devices with PC grating are capable of providing large scale of emitting apertures. So substrate emitting CRPC quantum cascade lasers will emit more light energy propagating along highly compressed spatial paths. Figure 5 shows the measured far-field intensity distributions for the substrate emitting device. The definition of the angular coordinates *θx* and *θy* in the far-field are corresponding to *x*- and *y*-axis in Figure 1a. At low injection current of 33.0 A (Figure 5a), a small divergence far-field was obtained and the full width at half maximum (FWHM) of the main lobe emission cone is *θx* × *θy* = 0.65° × 0.31°. At higher current levels (Figure 5b), an increased broadening of the main lobe (1.1° × 0.45°) is observed due to more pronounced thermal effects especially for broad area laser related to self-focusing effect [28]. Figure 5c shows the 1D far-field in *θx* direction derived from Figure 5a. The small dotted lines labeled TM_00_, TM_03_, and TM_06_ correspond to 1D fitting of the different order transverse far-field distribution. TM_03_ and TM_06_ is the third- and sixth-order transverse mode, respectively. In this direction, the far-fields exhibits hybrid transverse mode. Based on the measured 2D far-field intensity distributions (Figure 5a,b), we calculate the proportion of each transverse mode. At 33.0 A, TM_00_ weights with 35.7%, TM_03_ 34.7%, and TM_06_ 29.6%. At 53.3A, TM_00_ increased to 39.1%, TM_03_ down to 32.7%, and TM_06_ 28.2%. In addition, we find that the whole electric field intensity distribution in *θx* direction is slightly tilted by connecting a line of shoulder peak and main peak because the wave vector of $\overset{\rightharpoonup }{a_2}$ is oblique with $\overset{\rightharpoonup }{a_1}$ by an angle of 80°. The emergence of TM_03_ and TM_06_ indicates that their losses are insufficient to suppress the gain. Figure 5d shows the 1D far-field in *θy* direction derived from Figure 5a. The measurement far-field divergence angle in *θy* is much larger than diffraction-limited spreading angle 0.08°, which is mainly due to the real divergence angle which is too small for our measurement system to distinguish.

**Figure 6:**
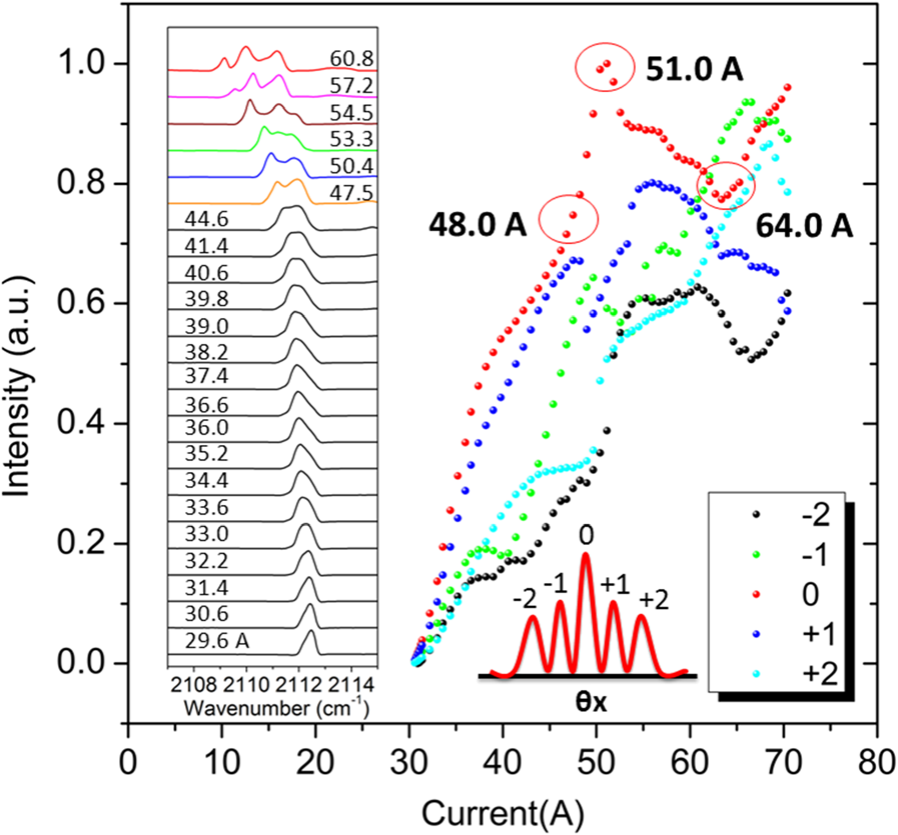
Intensity for +2, +1, 0, −1, and −2 order beam peaks under various injection current. From 1.05*I*th to 2.1*I*th at room temperature. Inset: Spectra of the CRPC substrate emitting QCL under an increased injection current from 29.6 to 60.8 A.

Mode competition in longitudinal and transverse direction strongly depends on driving the current [29]. In the far-field testing, we find an interesting phenomenon that the +2, +1, 0, −1, and −2 order beam peak intensities in *θx* direction are changing in a certain manner as the injection current increases, where the 0 order position correspond to TM_00_, and (+1,−1) pair corresponds to TM_03_ and (+2,−2) TM_06_. As shown in Figure 6, the 0 order and other high-order peak intensity increases as the driving current comes up to near 48 A. Then, the intensity of 0 order quickly rises to the maximum value as the driving current comes up to near 51.0 A. In contrast, +1 and −1 orders fall down to a low value while +2 and −2 orders jump up to a high value directly at the moment. This qualitatively reveals a transverse mode competition that TM_03_ is suppressed. Subsequently when the current continues to increase beyond 51.0 A, the TM_00_ begins to be weakened and the transverse far-field in *θx* direction exhibits a strong tendency towards higher mode distribution.

The left inset of Figure 6 shows the lasing spectra under pulsed operation at different current from 1.05*I*th to 2.1*I*th. The tested laser shows single longitudinal mode operation at low injection current level. When the injection current increases, the FWHM of spectrum widens from 0.44 cm^−1^ (29.6 A) to 1.16 cm^−1^ (44.6 A), and even muti-wavelength lasing (>46.0 A). This may be due to the nonuniform refractive index of 2D photonic-crystal DFB grating, resulting from inherent linear Gaussian-like distribution of heat in the active region, giving rise to a longitudinal inhomogeneous gain profile of different spectrum. It is much more obvious for a broad area device to suffer spectrum widening since the operation current is so high that more electric energy transform into heat. In the future, when a shorter pulsed width source is applied for the drive of QCL, the single longitudinal mode emission may persist to a higher operating current.

## Conclusions

In conclusion, we report a high power and small divergence substrate emitting centered rectangular photonic-crystal quantum cascade laser. A record maximum peak output power for vertical emitting QCLs exceeding 10 W is obtained. Divergence far-field angle below 1° (0.65° × 0.31°) for QCLs is first realized. And the main lobe of far-field occupies approximately 40% of output energy. The laser shows a strong single longitudinal mode operation around 4.73 μm with SMSR of 30 dB even at high temperature.

## Abbreviations

AR: anti-reflective
CRPC: centered rectangular photonic-crystal
CW: continuous wave
DEHL: double-exposure holographic lithography
DFB: distributed feedback
HR: high reflectivity
MCT: mercury cadmium telluride
PC: photonic-crystal
QCLs: quantum cascade lasers
SEM: scanning electron microscope
SE-QCLs: substrate emitting quantum cascade lasers
TM_00_: fundamental transverse mode
